# Erratum for: “Effect of JJYMD-C, a novel synthetic derivative of gallic acid, on proliferation and phenotype maintenance in rabbit articular chondrocytes *in vitro*” [Braz J Med Biol Res (2014) 47(8): e3935]

**DOI:** 10.1590/1414-431X20193935erratum

**Published:** 2019-07-10

**Erratum for:** Braz J Med Biol Res | doi: 10.1590/1414-431X20143935

The Authors would like to correct Figure 6 and Figure 8 that were published incorrectly in the article “Effect of JJYMD-C, a novel synthetic derivative of gallic acid, on proliferation and phenotype maintenance in rabbit articular chondrocytes *in vitro*” in volume 47 no. 8 (2014) of the *Brazilian Journal of Medical and Biological Research* <http://dx.doi.org/10.1590/1414-431X20143935>.

The images of cell viability of 0.125 µg/mL JJYMD-C at 2 days in Figure 6 and of immunohistochemical staining for type II collagen of control at 2 days in Figure 8 were mistakenly submitted. The correct Figures 6 and 8 are published below.

The authors apologize to the readers and to the Brazilian Journal of Medical and Biological Research.

**Figure 6.**
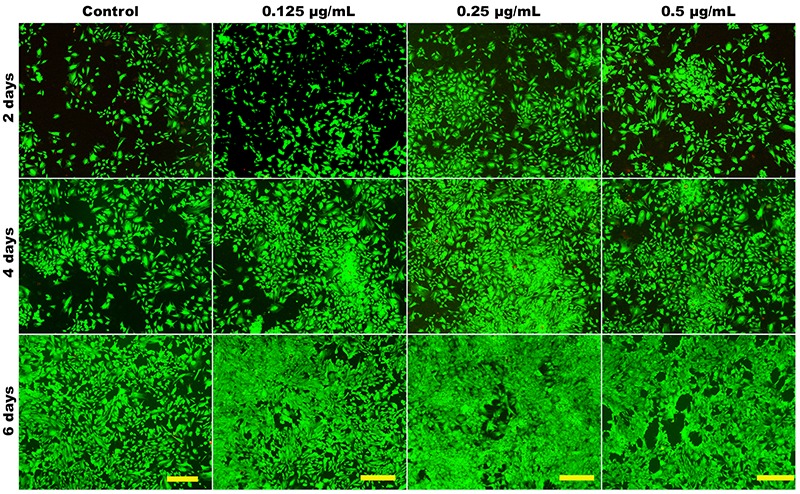
Laser-scanning confocal microscopy images showing the viability of chondrocytes cultured *in vitro* with 0 (control), 0.125, 0.25, and 0.5 mg/mL JJYMD-C for 2, 4, and 6 days. Scale bar: 100 mm.

**Figure 8.**
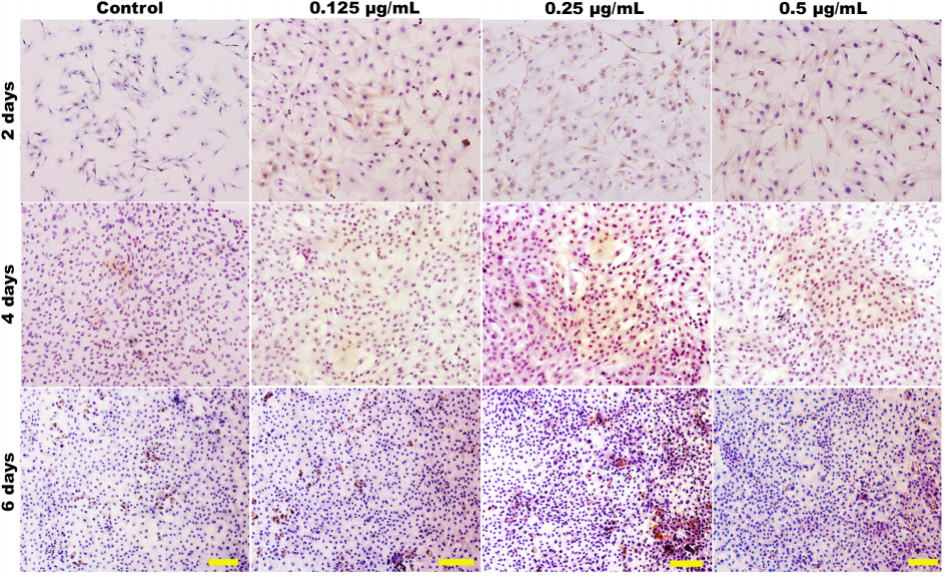
Immunohistochemical staining revealed the presence of type II collagen. Chondrocytes cultured *in vitro* with 0 (control), 0.125, 0.25, and 0.5 mg/mL JJYMD-C for 2, 4, and 6 days. Scale bar: 100 mm.

